# Validation of the Dutch version of the Edmonton Symptom Assessment System

**DOI:** 10.1002/cam4.3253

**Published:** 2020-07-09

**Authors:** Frederieke H. van der Baan, Josephine J. Koldenhof, Ellen J. de Nijs, Michael A. Echteld, Danielle Zweers, Ginette M. Hesselmann, Sigrid C. Vervoort, Jan B. Vos, Everlien de Graaf, Petronella O. Witteveen, Karijn P. Suijkerbuijk, Alexander de Graeff, Saskia C. Teunissen

**Affiliations:** ^1^ Center of Expertise in Palliative Care Julius Center for Health Sciences and Primary Care University Medical Center Utrecht University Utrecht The Netherlands; ^2^ Department of Medical Oncology Cancer Center University Medical Center Utrecht University Utrecht The Netherlands; ^3^ Center of Expertise Palliative Care Leiden University Medical Center Leiden The Netherlands; ^4^ Avans University of Applied Sciences Breda/Tilburg The Netherlands; ^5^ Cancer Center University Medical Center Utrecht University Utrecht The Netherlands

**Keywords:** cancer, patient reported outcome measures, quality of life, symptom assessment

## Abstract

**Background:**

The Utrecht Symptom Diary (USD) is a Dutch and adapted version of the Edmonton Symptom Assessment System, a patient‐reported outcome measurement (PROM) tool to asses and monitor symptoms in cancer patients. This study analyses the validity and responsiveness of the USD and the cutoff points to determine the clinical significance of a symptom score.

**Methods:**

Observational longitudinal cohort study including adult in‐ and outpatients treated in an academic hospital in the Netherlands who completed at least one USD as part of routine care (2012‐2019). The distress thermometer and problem checklist (DT&PC) was used as a reference PROM.

Content, construct and criterion validity, responsiveness, and cutoff points are shown with prevalences, area under receiver operating characteristic (ROC) curve, Chi‐squared test, Wilcoxon signed‐rank test, and positive and negative predictive values, respectively.

**Results:**

A total of 3913 patients completed 22 400 USDs. Content validity was confirmed for all added USD items with prevalences of ≥22%. All USD items also present on the DT&PC demonstrated a good criterion validity (ROC >0.8). Construct validity was confirmed for the USD as a whole and for the items dry mouth, dysphagia and well‐being (*P* < .0001). USD scores differed significantly for patients when improving or deteriorating on the DT&PC which confirmed responsiveness. Optimal cutoff points (3 or 4) differed per symptom.

**Conclusion:**

The USD is a valid 12‐item PROM for the most prevalent symptoms in cancer patients, which has content, criterion, and construct validity, and detects clinically important changes over time, in both curative and palliative phase.

## Graphical abstract

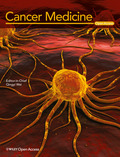

## 1 INTRODUCTION

Cancer patients experience many symptoms caused by their disease and/or its treatment which influence health‐related quality of life (HRQL). As healthcare professionals tend to underestimate symptoms and family members to over‐rate symptoms, symptoms reported by patients themselves are considered to be the most reliable indicators of symptom presence and intensity.^1^, ^2^, ^3^

Patient‐reported outcome measurement tools (PROMs) improve patient‐professional communication, reduction of symptom severity, reinforcement of patient autonomy, and patients’ satisfaction, regardless of the phase of their disease.^4^, ^5^, ^6^, ^7^, ^8^ Monitoring symptoms can reduce emergency department visits and improve predictions of life expectancy in terminal ill cancer patients.^9^, ^10^

Worldwide, a frequently used PROM to routinely asses and monitor symptoms in advanced cancer patients without anticancer treatment is the Edmonton Symptom Assessment System (ESAS).^11^ Since its development in 1991, the ESAS has been validated, translated, and adapted by multiple groups with a focus on advanced cancer inpatients. Reliability (test‐retest and inter‐rater reliability) and concurrent validity have been studied mostly, using a variety of other instruments to compare the ESAS to. Much less is known about the responsiveness and the cutoff points to distinguish patients with none, mild, moderate, and severe symptom burden.^4^, ^12^, ^13^

We developed an adapted Dutch version of the ESAS—the Utrecht Symptom Diary (USD)—which aims to support daily management of symptoms across the entire continuum of cancer care. In the last 20 years, the USD has been implemented in daily practice in several hospitals, general practices and hospices in the Netherlands. Moreover, usage of the USD is recommended by the Netherlands Quality Framework for Palliative Care.^14^

This study analyses the validity and responsiveness of the USD, as well as the cutoff points per symptom to determine the clinical significance of a symptom score.

## 2 METHODS

### 2.1 Participants

This observational longitudinal cohort comprises all adult cancer patients treated in the University Medical Center (UMC) Utrecht, the Netherlands, who completed at least one USD between August 2012 and July 2019. Within the UMC Utrecht Cancer Center filling out the USD is standard care, which means that each patient during each out‐patient treatment and each admission is asked to complete the symptom diary as a basis for tailoring care. However this does apply to patients with impaired cognitive behavior, not being able to read and understand Dutch language. As a result, no USD is available of these patients.

Some participants receive chemotherapy partly in the clinic and partly in the outpatient clinic. This research was not considered subject to the Medical Research Involving Human Subjects Act by the institutional review board of the UMC Utrecht. For each measurement property of the USD, we selected a subgroup of patients from this cohort.

### 2.2 Data collection

For the development of the USD, the ESAS items pain, fatigue, nausea, depression, anxiety, drowsiness, lack of appetite, shortness of breath, and feeling of well‐being were translated into Dutch. The items sleeping problems, dry mouth, abnormal stool, and dysphagia were added to the USD, based on their high prevalence in patients with incurable cancer.^15^, ^16^ Drowsiness was later excluded because of ambiguity in the Dutch language. The 12 items were clustered in two language specific sections starting with “I have...” and “I feel...” The ESAS item well‐being was translated as “I feel good’ to “I feel very bad” as the last summarizing question. Moreover, patients were able to add symptoms and to assign priority to symptoms which needed attention first, supporting patients’ autonomy. Symptoms were scored on a 0‐to‐10‐numeric rating scale (NRS), with higher values indicating increasing symptom intensity.

The USD (Appendix A) was offered daily to all inpatients, to assess and monitor their current symptoms and well‐being. Outpatients reported on symptoms and well‐being experienced *since the last visit to the outpatient clinic*. In addition, patients were offered the Distress Thermometer and Problem Checklist (DT&PC) at the start of a (new) treatment and when indicated. The Distress Thermometer and Problem Checklist is an internationally accepted and validated PROM recommended for early recognition of symptoms and detection of supportive care needs in cancer patients.^17^, ^18^ The Distress Thermometer asks patients to score their distress on a 0‐to‐10 visual analogue scale (VAS; a higher score indicating more distress). The Problem Checklist includes 35 items distributed over five domains: practical, family/social, emotional, spiritual, and physical problems. Patients score a symptom dichotomously, experiencing the symptom as a problem or not.

Patient characteristics, disease and treatment‐related data, and USD and DT&PC scores, were retrospectively collected from the electronic medical records.

### 2.3 Statistical analysis

Patient characteristics were summarized with descriptive statistics. Definitions, methods and quality criteria for the measurement properties of the USD were based on the COSMIN (Consensus‐based Standards for the selection of health Measurement INstruments) initiative.^19^, ^20^ Moreover, we analyzed cutoff points to determine the clinical significance of a symptom score. Patients with a missing value for the studied USD item were excluded for analysis for that item. R software for statistical computing and graphics v3.5.1 were used for statistical analysis.^21^

#### 2.3.1 Content validity

Content validity is defined as the extent to which the concepts of interest are represented by the USD items^20^. To study the patient’s perspective on the content, we asked a subgroup of 100 in‐ and outpatients in a questionnaire: “were the USD symptoms in line with your symptom burden?” A reported relevance by at least 80% of the patients was considered as a sufficient content validity. In addition, prevalence of the symptoms that were added in the USD to the symptoms of the original ESAS^11^ are presented to assess whether these items are relevant for our population.

#### 2.3.2 Criterion validity

Criterion validity is defined as the degree to which the USD scores are an adequate reflection of a “gold standard.”^19^ In the absence of a gold standard, for this analysis, the DT&PC was considered the reference standard.

For each patient, we selected the first DT&PC completed within 1 day of a USD. The items pain, sleeping problems, nausea, shortness of breath, fatigue, anxiety, and depressed mood are identical on both instruments and were used for the criterion validation. The USD item lack of appetite was compared to “problem with eating” on the DT&PC. The USD item abnormal stool was compared to the DT&PC diarrhea and constipation.

The area under the curve (AUC) of the receiving–operating curve (ROC) was calculated using the USD scores as predictive values and the DT&PC as the reference standard, indicating the “true” condition. An AUC of at least 0.70 was considered positive for the criterion validity.^22^ The corresponding 95% confidence intervals were computed with 2000 stratified bootstrap replicates.^23^

#### 2.3.3 Construct validity

Construct validity is defined as the extent to which USD scores are consistent with theoretically derived hypotheses.^19^ Prior to the analyses we formulated one overall hypothesis concerning all USD items and three hypotheses for the items that are not part of the DT&PC, dry mouth, dysphagia, and well‐being:

- “The prevalence and intensity of all symptoms will increase with progression of disease.”^15^, ^24^ Inpatients were divided into two disease stages: inpatients receiving chemotherapy, either with curative or palliative intent, and inpatients receiving symptom directed palliation only. Outpatients and patients admitted for other reasons than chemotherapy treatment were excluded, as we could not determine their disease stage with certainty. The first completed USD during the first hospital admission was used to compare symptom prevalence and intensity.
- “Patients using opioids^25^ experience dry mouth more often than patients who do not use opioids.” The first USD of each inpatient was selected, due to the availability of a complete medication list. USD scores for dry mouth were compared in patients using and patients not using opioids.
- “Patients with head and neck cancer (HNC) experience dysphagia more often than patients with other cancer diagnoses.”^26^ The first USD of HNC patients was compared to the first USD of patients with other primary diagnoses.
- “Patients with pain report poorer well‐being than patients without pain.”^27^ For this purpose, we compared well‐being on the first USD of all patients reporting a pain score ≥3 with patients reporting a pain score <3.

In the literature, the optimal cutoff point of the ESAS items remains unclear and varies from 2 to 5 for symptom presence and moderate symptom intensity.^28^, ^29^, ^30^ In previous research, we found that HRQL decreased due to the experience of multiple symptoms with scores <3 at the same time.^31^, ^32^, ^33^ Therefore, we considered a USD score ≥3 as clinically relevant. For all hypotheses, we compared the prevalence of a clinically relevant symptom (USD score <3 vs ≥3) and intensity (median score) using a chi‐squared test and the nonparametric Mann‐Whitney *U* test, respectively. For the USD item well‐being, only the intensity was compared, since dichotomization of well‐being was not considered to be meaningful.

#### 2.3.4 Responsiveness

Responsiveness is defined as the ability of the USD to detect change over time, a measure of longitudinal validity.^19^, ^22^ We selected patients with two subsequent DT&PCs completed within 1 day of a USD. Per USD item patients were selected who “improved” (reporting a problem on the first DT&PC and no problem on the second) or “deteriorated” (no problem on the first DT&PC and a problem on the second). For each USD item, we compared the median USD score at both measurement points, using Wilcoxon signed‐rank test.

#### 2.3.5 Cutoff points

By using the USD score and the corresponding problem on the DT&PC, the cutoff point on the USD that best discriminates between patients with and without a clinically significant symptom score was assessed. We selected the first DT&PC that was completed within one day of a USD of all patients. For each item, we explored the performance of cutoff points of 2, 3, and 4 in terms of positive predictive value (PPV) and negative predictive value (NPV), predicting for the presence or absence of the corresponding problem on the DT&PC, respectively.

## 3 RESULTS

A total of 3913 unique patients with cancer completed over 22 400 USDs. Patient characteristics at the time of the first available USD are presented in Table 1, for the whole group, by thepresence of concurrent DT&PC and by disease stage.

**TABLE 1:** Patient characteristics

	Total		USD and DT&PC within one day		Chemotherapy (curative and palliative)		Symptom‐directed palliation only	
	N = 3913		N = 1353		N = 1919		N = 224	
Age ‐ mean (SD)	60.6	(13)	59.6	(12)	58.2	(13)	63.4	(12)
Gender male—N (%)	2197	(56)	741	(55)	1027	(54)	125	(56)
Patient								
Outpatients—N (%)	1689	(43)	1101	(81)	1147	(60)	2	(1)
Inpatients—N (%)	2215	(57)	248	(18)	769	(40)	222	(99)
Missing—N (%)	9	(<0.1)	4	(<0.1)	3	(<0.1)	NA	NA
Duration admission (days) —median [IQR]	7	[5‐14]	5	[4‐7]	6	[4‐8]	10	[6‐14]
Primary cancer site, N (%)								
Digestive tract	1232	(31)	439	(32)	627	(33)	105	(47)
Bone marrow and lymph nodes	549	(14)	66	(5)	194	(10)	2	(1)
Female genital tract	370	(9)	184	(14)	327	(17)	25	(11)
Head and neck	322	(8)	143	(11)	284	(15)	11	(5)
Central nervous system	255	(7)	39	(3)	60	(3)	1	(0)
Male genital tract	250	(6)	107	(8)	178	(9)	16	(7)
Breast	245	(6)	105	(8)	151	(8)	20	(9)
Skin	244	(6)	143	(11)	24	(1)	24	(11)
Kidney and urinary tract	180	(5)	42	(3)	34	(2)	10	(4)
Lung and mediastinum	168	(4)	65	(5)	11	(1)	1	(0)
Endocrine	43	(1)	5	(0)	9	(0)	4	(2)
Other	55	(1)	15	(1)	20	(1)	5	(2)
ECOG PS, N (%)								
0‐1	1430	(37)	595	(44)	1024	(53)	21	(9)
2	236	(6)	60	(4)	128	(7)	24	(11)
3‐4	123	(3)	8	(1)	25	(1)	42	(19)
Missing	2124	(54)	690	(51)	742	(39)	137	(61)
Days between PS and USD ‐ median [IQR]	6	[1‐14]	5	[0‐13]	6	1‐14]	5	[1‐14]

Abbreviations: DT&PC, Distress Thermometer and Problem Checklist; ECOG PS, Eastern Cooperative Oncology Group performance score; IQR, interquartile range; NA, not applicable; SD, standard deviation; USD, Utrecht Symptom Diary.

The subgroup of patients with a concurrent DT&PC consisted mainly of outpatients (81%). Sixty percent of the patients received chemotherapy as an outpatient. Nearly all patients receiving symptom directed palliation only were admitted to the hospital, with a median stay of 10 days. Data on Eastern Cooperative Oncology Group (ECOG) performance status (PS) were available for 46% of patients.

### 3.1 Content validity

A total of 100 patients, 72% inpatients and 28% outpatients, completed the study specific questionnaire. 86% answered that the USD items properly represented their symptom burden. The prevalence of sleeping problems, dry mouth, abnormal stool, and dysphagia are shown in Table 2 for the total study population. The prevalence of ≥22% show the importance of these items, confirming content validity of these added USD items.

**TABLE 2:** Content validation

	Prevalence USD score ≥3 N (%)
Sleeping problems (N = 3801)	1640 (43)
Dry mouth (N = 3497)	1491 (43)
Abnormal stool (N = 3698)	1629 (44)
Dysphagia (N = 3485)	755 (22)

Abbreviation: USD, Utrecht Symptom Diary.

### 3.2 Criterion validity

A total of 1353 patients (35%) completed at least once a USD and DT&PC within 1 day. 82% of the inpatients who filled out a DT&PC completed it on the first admission day and 18% on another day during admission. For all items, the percentage of missing values was ≤3.5%. See Table 3 for results on criterion validity, comparing the USD scores to the dichotomous outcome of the DT&PC. The lowest AUC is 0.8, demonstrating good criterion validation.

**TABLE 3:** Criterion validity

	USD and DT&PC (N)	Prevalence on DT&PC (%)	AUC [95% CI]
Pain	1332	35.2	0.91 [0.89‐0.93]
Sleeping problems	1329	32.1	0.90 [0.88‐0.92]
Anorexia	1323	27.4	0.81 [0.79‐0.84]
Abnormal stool	1305	25.4	0.89 [0.87‐0.91]
Nausea	1330	13.9	0.91 [0.88‐0.94]
Dyspnea	1325	12.1	0.93 [0.91‐0.95]
Fatigue	1332	50.5	0.91 [0.89‐0.92]
Anxiety	1326	27.8	0.87 [0.85‐0.89]
Depressed mood	1316	22.7	0.87 [0.85‐0.90]

Abbreviations: AUC, Area under ROC curve; CI, confidence interval; DT&PC, Distress Thermometer and Problem Checklist; USD, Utrecht Symptom Diary.

### 3.3 Construct validity

A total of 1919 patients (49%) completed a USD during chemotherapy, and 224 (6%) when receiving symptom‐directed palliation only. Table 4 summarizes symptom prevalence. During chemotherapy every symptom—except for dyspnea—occurred in >10% of the patients. Highest scores were found for fatigue. During the phase of symptom‐directed palliation only, every symptom occurred in ≥25%. A median score of ≥3 was found for 8/12 items. Again, fatigue had the highest intensity. Both the prevalence of USD scores ≥3 and the median scores were higher for all symptoms in patients receiving symptom directed palliation only than in patients during chemotherapy. Thus, the first hypothesis, stating that the prevalence and intensity of all symptoms increase with progression of disease, is confirmed, demonstrating the construct validation for all USD symptom items.

**TABLE 4:** Construct validity—Hypothesis 1 “Prevalence and intensity of all symptoms will increase with progression of disease”

	Prevalence—N (%) USD score ≥3		*P*‐value^a^	Intensity— median [IQR]		*P*‐value^b^
	Chemotherapy N = 1919	Symptom directed palliation only N = 224		Chemotherapy N = 1919	Symptom directed palliation only N = 224	
Pain	452 (24)	100 (45)	<.0001	1 [0‐3]	3 [1‐6]	<.0001
Sleeping problems	539 (29)	117 (53)	<.0001	1 [0‐4]	4 [1‐7]	<.0001
Dry mouth	466 (25)	155 (70)	<.0001	0 [0‐3]	5 [2‐8]	<.0001
Dysphagia	318 (17)	62 (28)	<.0001	0 [0‐2]	1 [0‐4]	<.0001
Anorexia	614 (33)	153 (72)	<.0001	1 [0‐5]	6 [3‐8]	<.0001
Abnormal stool	548 (30)	131 (64)	<.0001	1 [0‐5]	5 [2‐8]	<.0001
Nausea	232 (12)	55 (25)	<.0001	0 [0‐1]	0 [0‐3]	<.0001
Dyspnea	141 (8)	69 (31)	<.0001	0 [0‐1]	1 [0‐4]	<.0001
Fatigue	779 (41)	165 (74)	<.0001	3 [0‐5]	6 [3‐8]	<.0001
Anxiety	356 (19)	77 (36)	<.0001	0 [0‐3]	1 [0‐5]	<.0001
Depressed mood	308 (17)	79 (37)	<.0001	1 [0‐3]	3 [1‐6]	<.0001
Well‐being	*NA*	*NA*		1 [0‐4]	4 [1‐7]	<.0001

Abbreviations: IQR, inter quartile range; NA, not applicable.

^a^ Chi square.

^b^ Mann‐Whitney U

As shown in Table 5, hypotheses 2‐4 were confirmed, showing construct validity of the items dry mouth, dysphagia, and well‐being, respectively.

**TABLE 5:** Construct validity—Hypotheses 2‐4

	N	USD score ≥3 N (%)	*P*‐value^a^	Intensity median [IQR]	*P*‐value^b^
Dry mouth					
Opioid use	537	309 (58)	<.0001	4 [2‐7]	<.0001
No opioid use	1678	616 (37)		2 [0‐5]	
Dysphagia					
Head and neck	322	117 (37)	<.0001	2 [0‐5]	<.0001
Other diagnoses	3591	475 (15)		0 [0‐2]	
Well‐being					
Pain score ≥3	1402	NA	NA	5 [3‐6]	<.0001
Pain score <3	2506	NA		2 [0‐4]	

Abbreviations: IQR, inter quartile range; NA,not applicable.

^a^ Chi square,

^b^ Mann‐Whitney U.

### 3.4 Responsiveness

A total of 293 patients (7%) completed >1 DT&PC and USD within 1 day. The vast majority (>80%) are outpatients as in our clinical setting the DT&PC is mostly offered to outpatients. Table 6 shows median scores (IQR) before and after symptom improvement or deterioration. The measurements were on average 42 days apart [IQR 7‐122]. For all items, the median USD score upon improvement is lower on T2 than on T1 and vice versa upon deterioration. For both improvement and deterioration, median change was 3.

**TABLE 6:** Responsiveness

	Improvement			*P*‐value^a^	Deterioration			*P*‐value^a^
	N	Median score [IQR]			N	Median score [IQR]		
		T1	T2			T1	T2	
Pain	41	3 [2‐5]	2 [0‐3]	.0028	59	0 [0‐2]	4 [2‐6]	<.0001
Sleeping problems	51	4 [2‐7]	1 [0‐2]	<.0001	53	0 [0‐2]	3 [2‐5]	<.0001
Anorexia	45	5 [2‐7]	2 [0‐5]	.011	39	1 [0‐5]	5 [3‐7]	<.0001
Abnormal stool								
Diarrhea	23	5 [2‐6]	2 [0‐3]	.015	36	1 [0‐3]	5 [2‐6]	<.0001
Obstipation	26	5 [4‐6]	2 [1‐5]	.005	46	0 [0‐1]	5 [3‐7]	<.0001
Nausea	24	3 [2‐5]	1 [0‐2]	.00069	50	0 [0‐1]	3 [1‐4]	<.0001
Dyspnea	13	3 [2‐4]	0 [0‐2]	.019	28	0 [0‐0]	3 [2‐5]	<.0001
Fatigue	38	4 [3‐5]	2 [1‐3]	.00016	70	1 [0‐3]	4 [2‐5]	<.0001
Anxiety	31	3 [2‐5]	1 [0‐2]	<.0001	35	0 [0‐2]	3 [2‐5]	<.0001
Depressed mood	21	4 [3‐5]	1 [0‐3]	<.0001	42	0 [0‐1]	3 [2‐5]	<.0001

T1=first concurrent DT&PC and USD; T2=subsequent concurrent DT&PC and USD.

^a^ Wilcoxon test.

### 3.5 Cutoff points

Table 7 shows the performance of three different cutoff points (2, 3, and 4) per item on the USD 0‐10 NRS. As expected for all items a lower cutoff increases the NPV. For a cutoff point of ≥3, NPV varied from 0.84 (fatigue) to 0.96 (dyspnea) and for a cutoff ≥4 from 0.75 (fatigue) to 0.95 (dyspnea). For both cutoff scores fatigue, pain, and anxiety had the lowest NPV’s.

**TABLE 7:** Performance of different cutoff points for the USD items

	Cutoff point USD					
	≥2		≥3		≥4	
	PPV (95%CI)	NPV (95%CI)	PPV (95%CI)	NPV (95%CI)	PPV (95%CI)	NPV (95%CI)
Pain	0.72 (0.68, 0.75)	0.94 (0.92, 0.96)	0.79 (0.75, 0.83)	0.88 (0.86, 0.90)	0.85 (0.81, 0.89)	0.81 (0.78, 0.83)
Sleeping problems	0.67 (0.63, 0.71)	0.95 (0.93, 0.96)	0.74 (0.70, 0.78)	0.90 (0.87, 0.92)	0.83 (0.79, 0.87)	0.84 (0.81, 0.86)
Anorexia	0.52 (0.48, 0.57)	0.90 (0.88, 0.92)	0.59 (0.54, 0.63)	0.89 (0.87, 0.91)	0.64 (0.59, 0.69)	0.87 (0.84, 0.89)
Abnormal stool	0.56 (0.52, 0.60)	0.95 (0.93, 0.97)	0.65 (0.60, 0.70)	0.92 (0.90, 0.94)	0.71 (0.66, 0.76)	0.89 (0.87, 0.91)
Nausea	0.62 (0.56, 0.68)	0.96 (0.95, 0.97)	0.77 (0.70, 0.83)	0.95 (0.93, 0.96)	0.85 (0.77, 0.91)	0.93 (0.91, 0.94)
Dyspnea	0.59 (0.52, 0.65)	0.98 (0.97, 0.99)	0.69 (0.61, 0.76)	0.96 (0.95, 0.97)	0.79 (0.71, 0.86)	0.95 (0.93, 0.96)
Fatigue	0.77 (0.74, 0.80)	0.93 (0.90, 0.95)	0.83 (0.80, 0.86)	0.84 (0.81, 0.87)	0.89 (0.87, 0.92)	0.75 (0.71, 0.78)
Anxiety	0.64 (0.60, 0.69)	0.92 (0.90, 0.93)	0.76 (0.71, 0.80)	0.88 (0.86, 0.90)	0.80 (0.75, 0.85)	0.84 (0.81, 0.86)
Depressed mood	0.55 (0.50, 0.60)	0.94 (0.92, 0.96)	0.68 (0.62, 0.73)	0.90 (0.88, 0.92)	0.79 (0.73, 0.84)	0.87 (0.85, 0.89)

Abbreviations: CI, confidence interval; NPV, negative predictive value; PPV, positive predictive value.

## 4 DISCUSSION

Previous validations of the ESAS have mainly focused on reliability and concurrent validity in advanced cancer inpatients, using a variety of other instruments to compare the ESAS to. Relatively less evidence is available on responsiveness and cutoff points.^4^, ^12^, ^13^ Our study fills part of this gap, as we show that the USD, a Dutch and adapted version of the ESAS, is a valid PROM for the most prevalent symptoms in cancer patients within all stages of disease. We also show the content validity of the added items to the USD and the ability to detect clinically important changes over time (responsiveness). Finally, we provide information about the clinical consequences of the generally used cutoff points.

### 4.1 Content validity

Our results show content validity of all measured items as patients reported them to reflect their symptom burden. The usefulness of our newly added items—sleeping problems, dry mouth, abnormal stool, and dysphagia—is confirmed since they occur in 22%‐44% of our population. Adding items to the ESAS has occurred before but not specifically in cancer patients.^34^, ^35^ Besides synonyms have been used for items such as constipation and sleep‐related problems.^36^, ^37^ Although dry mouth is not part of the ESAS, it is part of the MD Anderson Symptom Inventory (MDASI), which also is a validated and frequently used PROM in cancer patients.^38^ The identification of dysphagia as a symptom to predict life expectancy by Teunissen et al^15^ has been endorsed by others^39^, emphasizing the relevance of including this item.

### 4.2 Criterion validity

We found a good concurrent criterion validation of the USD items pain, sleeping problems, anorexia, abnormal stool, nausea, dyspnoea, fatigue, anxiety, and depressed mood, using the dichotomous outcome of the DT&PC. This means the USD is a valid instrument to reflect symptom burden at the time of assessment as well as over a previous period of time. Previous studies have investigated concurrent criterion validity of translated and/or modified versions of the ESAS with other PROMs, as reviewed in detail^4^, ^13^, also concluding that these ESAS versions are valid for symptom assessment in different palliative care settings.^37^, ^40^, ^41^ The strength of our study is that the USD uses a NRS and the DT&PC questions whether a symptom was considered a problem, therefore reflecting the patient’s perspective on symptom scores. Consequently, insight into patients’ personal cutoff point can be obtained. In previous studies, this was not possible since the PROMs used for comparison both utilized measuring scales. Hui and Bruera^12^ reflected on this importance by describing how one patient may consider a score of 6/10 as agonizing while another may find it acceptable.

We used routine clinical data, which may be a limitation of our study. Since we only have information on the DT&PC when it was offered and completed, selection bias may be implied. Second, the DT&PC asks patients to report on symptoms over a time window of a week, whereas the USD captures current symptoms for inpatients and symptoms since last visit for outpatients. In our population, 82% of inpatients completed the USD and DT&PC on the first admission day. This makes it very likely that the symptom burden represents the patient’s situation of the days before the admission as well.

### 4.3 Construct validity

We found a good construct validity on the USD items dry mouth, dysphagia, and well‐being. To the best of our knowledge, there is only one other study using hypothesis testing to validate a translated and modified ESAS version in a small convenience sample of 23 cancer patients.^42^ Several groups studied construct validity by investigating correlations between clusters of symptoms, hypothesizing a larger underlying construct measured by the ESAS items.^12^, ^43^ However, we decided to consider each symptom as an independent “construct.” A sum score of all items has been studied^41^, ^44^, as suggested by Bruera et al^11^ to represent overall symptom distress as a construct. As we question the underlying assumption that low USD scores on multiple symptoms is comparable to a single high symptom score, based on other work of our group^31^, we decided not to summarize scores in this study.

### 4.4 Responsiveness

Our results on responsiveness show that the USD is able to detect clinically significant differences over time for all items, as we show that patients who report improvement or deterioration on items of the DT&PC, have lower and higher USD scores at the second time point, respectively.

Paiva et al^45^ studied responsiveness of the Brazilian version of the ESAS using an anchor‐based method, asking 80 patients to classify after 21 days whether their symptoms were worse, the same or better than experienced during the first visit. Although they found that the median scores of patients who felt better indeed improved, and those of patients who reported a worsened condition were decreased, they could not show responsiveness for all items. Most probably this was caused by their small sample size and a patient population with a low symptom burden.

The strength of our comparison of the USD to a concurrent DT&PC is that we compared USD scores to a reflection by the patients of the symptom as a problem or not, which makes improvement or deterioration of symptoms clinically relevant. We did not find evidence that in‐ and outpatients score differently on the USD when a symptom improved or deteriorated according to the DT&PC. Moreover, we performed analyses with multiple measures of one individual patient in order to obtain criterion validity, construct validity, and responsiveness data. Patient setting will not likely influence these within‐person analyses.

A limitation of our approach is that we do not know which patients remained stable, since reporting a symptom on both T1 and T2 as a problem on the DT&PC does not inform us whether the patient experienced this symptom in the same way at both moments. Consequently, calculating an AUC, which is the measure for responsiveness^22^, as well as the minimal clinically important difference for improvement and deterioration was not possible. The latter was studied by Hui et al for the ESAS^46^, concluding that for all symptoms the optimal cutoff for improvement was ≥1 point and ≤1 point for deterioration. Though with sensitivities of only 59%‐85%, indicating relatively many false negatives, which are patients who actually experienced a symptom change, but are missed with these cutoffs.

### 4.5 Cutoff points

The symptoms of the ESAS with scores of 0, 1‐3, 4‐6, and 7‐10 are generally considered as absent, mild, moderate, and severe, respectively.^29^, ^47^, ^48^ However, we found that, when using NRS ≥4 as cutoff for moderate symptom burden, except for nausea and dyspnoea, >10% of patients with a score <4 would be misclassified as having “none” or “mild” symptoms, whereas in fact they reported the symptom as a problem. It is likely that in certain circumstances and for certain items other cutoffs should be used, which also is suggested by Hui et al.^49^ Later on in the disease process, patients in our cohort seem to accept a higher symptom burden which endorses the findings of Dalal et al^50^ who found that patients with advanced disease reported to pursue a pain score of 3. By using different cutoffs depending on the situation and personal goals of the individual patient, a more person‐centered approach may be achieved, improving shared decision‐making^49^.

In conclusion, our results illustrate that the USD is a valid 12‐item PROM containing the most prevalent symptoms in cancer patients. The USD has proven content, criterion and construct validity and can detect clinically important symptom changes over time in both in‐ and outpatients through the whole continuum of cancer care. As a result the USD improves insight into symptom burden in the individual patient which is essential for comprehensive personalized care in daily oncology practice.

## CONFLICT OF INTEREST

JK: advisory role Novartis (paid to institution). KS: advisory role Roche, Novartis, MSD, BMS, Pierre Fabre (all paid to institution). The other authors have no conflict of interest to declare.

## AUTHOR CONTRIBUTIONS

FB, JK: Conceptualization, Data curation, Formal analysis, Investigation, Methodology, Project administration, Resources, Validation—Verification, Visualization, Writing original draft. EN, ME: Conceptualization, Investigation, Validation—Verification. DZ, GH, SV, JV, EG, KS: Writing—review and editing. PW: Supervision , Writing—review and editing. AG: Conceptualization, Investigation, Methodology, Validation—Verification, Writing—review and editing, Visualization. ST: Conceptualization, Investigation, Methodology, Resources, Validation—Verification, Supervision, Visualization.

## Supporting information

Supinfo

## Data Availability

The data that support the findings of this study are available from the corresponding author upon reasonable request.
