# Supplementary material for: Validation of the Dutch version of the Edmonton Symptom Assessment System
Source: Cancer Med. 2020 Jul 9;9(17):6111–21. doi: 10.1002/cam4.3253 (PMC7476846; doi:10.1002/cam4.3253)
Supplement: Supplementary file 1 — Supinfo [file CAM4-9-6111-s001.docx]

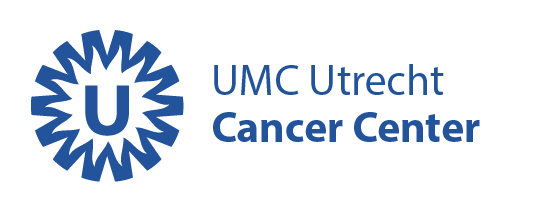


| **Utrecht Symptom Diary (USD)**  *Core instrument*  **Date ….../……./……..** | | |
| --- | --- | --- |
| **Instruction**  By reporting symptoms on a daily basis together we can plan the appropriate care for you, and evaluate and adjust support when necessary. Could you please circle - per symptom - the number that best describes how much burden you experience at the time of completion? We also ask you ‘how you feel‘ with the item 'well-being'.  If you suffer from symptoms and/or feelings that are not listed, please indicate them in the extra lines. | | |
| **I have/feel** | | |
| no pain | 0 1 2 3 4 5 6 7 8 9 10 | worst possible pain |
| no sleeping problems | 0 1 2 3 4 5 6 7 8 9 10 | worst possible sleeping problems |
| no dry mouth | 0 1 2 3 4 5 6 7 8 9 10 | worst possible dry mouth |
| no dysphagia | 0 1 2 3 4 5 6 7 8 9 10 | worst possible dysphagia |
| no lack of appetite | 0 1 2 3 4 5 6 7 8 9 10 | worst possible lack of appetite |
| no abnormal stool | 0 1 2 3 4 5 6 7 8 9 10 | worst possible abnormal stool |
| no nausea | 0 1 2 3 4 5 6 7 8 9 10 | worst possible nausea |
| no shortness of breath | 0 1 2 3 4 5 6 7 8 9 10 | worst possible shortness of breath |
| no fatigue | 0 1 2 3 4 5 6 7 8 9 10 | worst possible fatigue |
| no anxiety | 0 1 2 3 4 5 6 7 8 9 10 | worst possible anxiety |
| no depressed mood | 0 1 2 3 4 5 6 7 8 9 10 | worst possible depressed mood |
|  |  |  |
| best feeling of well-being | 0 1 2 3 4 5 6 7 8 9 10 | worst possible feeling of well-being |
| **Other symptom(s)** |  |  |
| ……………………………………. | 0 1 2 3 4 5 6 7 8 9 10 | ……………………………… |
| ……………………………………. | 0 1 2 3 4 5 6 7 8 9 10 | ……………………………… |
| ……………………………………. | 0 1 2 3 4 5 6 7 8 9 10 | ……………………………… |

| **Which symptom(s) bothers you the most and is your priority for support?** |
| --- |
|  |

©2016 MOD/UMC Utrecht Cancer Center
